# The effects of blueberry (*Vaccinium corymbosum* L.) and jujube fruit (*Ziziphus jujube*) on physicochemical, functional, and sensorial properties, and probiotic (*Lactobacillus acidophilus* DSM 20079) viability of probiotic ice cream

**DOI:** 10.1002/fsn3.3955

**Published:** 2024-01-09

**Authors:** Gülsüm Şentürk, Nihat Akın, Çiğdem Konak Göktepe, Begüm Denktaş

**Affiliations:** ^1^ Department of Food Engineering, Faculty of Agriculture Selcuk University Konya Turkey

**Keywords:** blueberry, functional food, ice cream, jujube fruit, *Lactobacillus acidophilus*, probiotic

## Abstract

The effects of blueberry (BB) and jujube fruit (JF) on the quality parameters, functional, probiotic (*Lactobacillus acidophilus* DSM 20079) viability, and sensorial properties of probiotic ice cream were investigated. No statistical differences were discovered regarding titratable acidity and *L. acidophilus* DSM 20079 counts between all samples. However, the ice creams preserved the survivability of probiotic bacteria during the storage period. The probiotic ice creams had counts of viable *L. acidophilus* DSM 20079 ranging from 8.42 to 8.80 log CFU/g which met the minimum required to achieve probiotic effects after 60 days of storage. Probiotic ice cream with BB or JF had significantly lower L* values than the control, and the BB addition caused the greatest decrease. The addition of both fruits clearly enhanced the total phenolic content and antioxidative activity in ice cream. The incorporation of BB or JF into the ice creams did not statistically affect the overrun value, while the addition of both fruits dramatically affected the first dripping time and increased hardness. Overall, sensory attributes were not significantly altered by the fortification of either fruit relative to the control, so these fruits can be added at higher concentrations to ice cream formulations for further studies.

## Graphical abstract

The effects of blueberry (BB) and jujube fruits (JF) on ice cream were examined. BB and JF improved the total phenolic content and antioxidant activity of samples. The ice cream added BB and JF had similar general acceptance scores with the control.

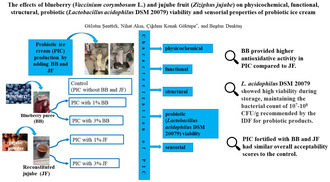

## 1 INTRODUCTION

Ice cream is a three‐phase system consisting of fat globules, ice crystals, and air bubbles surrounded by a freeze‐concentrated aqueous or matrix phase that involves the proteins, sugars, and salts (Goff, 2003). Ice cream is conventionally produced milk‐based and can improve the formulation with fruits, seeds, and functional compounds such as probiotics, prebiotics, and natural antioxidants (Haghani et al., 2021). The addition of pulp, juices, pomace, and extracts of various fruits to ice cream has gained popularity due to the increasing consumer desire for functional and fruit‐flavored ice cream (Kavaz Yuksel, 2015).

Blueberry is an excellent supplier of natural bioactive components phenolics, vitamin C, anthocyanin, and flavonoids (Wang et al., 2017). Blueberries have become very popular among consumers in many studies as a functional food with various activities, including antioxidant (Pertuzatti et al., 2014), antibacterial (Heinonen, 2007), antiviral (Joshi et al., 2016), resistance to cardiovascular disease (Liu et al., 2016), immunity enhancement and anticancer (Folmer et al., 2014). Therefore, in many studies, blueberry was added to ice creams as a fruit with a high processing potential, and the functional and technological properties of these products were investigated (Kotan, 2018; Sayar et al., 2022; Tarakçı & Durak, 2020; Turan et al., 2015).

Jujube is another delicious fruit known for its numerous health benefits that is widely grown in subtropical and tropical regions, particularly in North Africa, Middle Eastern, and East Asia countries. Scientific studies have shown that jujube fruits have various nutritional and functional ingredients including polysaccharides, amino acids, triterpenoid acids, flavonoids, phenolic acids, and mineral components (Rashwan et al., 2020). These phytocomponents of jujube exhibit antioxidant, anticancer, anti‐inflammatory, gastrointestinal‐protective, immunostimulant, and neuroprotective effects (Guo et al., 2015). In the literature, it is seen that quite detailed studies have been done on the chemical composition, biological activities, bioactive compounds, and preservation methods of jujube (Guo et al., 2016; Li et al., 2009; Sun et al., 2019). In addition, jujube has been added to different food formulations and innovative jujube‐based foods have been researched. These products include sponge cake (Najjaa et al., 2020), vinegar (Vithlani & Patel, 2010), fermented fruit juice (Cai et al., 2019), wine (Zhang et al., 2016), and juice (Zhao et al., 2016). However, these limited numbers of food products should be increased, and jujube should be added to different food formulations to improve product quality. In this study, it is aimed to create a potential application for the commercialization of this fruit by adding jujube to probiotic ice cream in order to benefit from its functional properties.

In addition to being a food with high nutritional quality, substantial proteins, and valuable fat, ice cream is a good carrier for probiotic cultures (Cruz et al., 2009). In the literature, many probiotic strains have been used in ice cream formulations and their viability has been investigated (Çam et al., 2023; Elkot et al., 2022; Sabet‐Sarvestani et al., 2021). Moreover, *L. acidophilus* DSM 20079 has previously been used successfully in the manufacturing of probiotic ice cream to benefit from its gastrointestinal and digestive health and therapeutic properties (Ergin et al., 2016; Linn et al., 2019).

The aim of this research was to evaluate how different levels of blueberry (BB) and jujube fruit (JF) affect the physicochemical, technological, and functional atributes of probiotic ice cream. Besides, the viability of probiotic *L. acidophilus* DSM 20079 was monitored during storage at −18°C for 60 days.

## 2 MATERIALS AND METHODS

### 2.1 Materials

Whole cow's milk was supplied by the Selcuk University Agriculture Faculty farm (Konya, Türkiye). The skim milk powder (96% non‐fat milk solids) and cream (42% of milk fat) were obtained from ENKA Dairy Company (Konya, Türkiye). Sahlep, sucrose, egg yolk, and guar gum were purchased from a local supplier in Konya, Türkiye. Blueberries (*Vaccinium corymbosum* L.) were supplied from the local market in in Konya, Türkiye. JF (*Ziziphus jujube*) powder was purchased in Isparta, Türkiye. JF powder was obtained by convection drying at 45°C for 20 h and ground to a fine powder. *L. acidophilus* DSM 20079 was provided from culture collection of Selcuk University, Department of Food Engineering (Konya, Türkiye).

### 2.2 Preparation of blueberries and jujube fruit puree

The fresh BB fruits were washed and left for a few minutes until the washing water drained. Cleaned blueberries were pureed with a blender and kept at −18°C until used.

The commercial hot air convective drying JF powder was reconstituted in 12% total solid content with water and then mixed using an Ultra Turrax mixer (IKA, Merck, Germany) until all ingredients were dissolved in water. The liquid blend was heated up 40°C in the water bath and 0.5% ĸ‐carrageenan (Sigma‐Aldrich, Louis, Missouri, USA; w/v) was added into reconstituted powder to form gel structure. The obtained mixtures were transferred to glass jar and pasteurized at 80°C for 1 min. The prepared JF puree was stored at 4°C until used.

### 2.3 Preparation of probiotic bacteria and growing conditions

The glycerol stock culture of *L. acidophilus* DSM 20079 was activated to get 10^8^ CFU/mL of viable cells in MRS (de Man, Rogosa, Sharpe) broth (Merck, Darmstadt, Germany) at 37°C for 24 h under aerobic conditions. The probiotic cells were harvested by centrifuging the biomass at 4500*g* for 30 min at 4°C and washed three times with sterile peptone solution (0.1%, w/v). The viable cell counts of *L. acidophilus* DSM 20079 in prepared bacterial suspension were 10^8^ CFU/mL.

### 2.4 Probiotic ice cream production

Ice cream samples were manufactured at pilot plant in the Department of Food Engineering at Selcuk University (Konya, Türkiye). The raw whole cow's milk was preheated to 45°C and mixed with calculated amounts of cream. Other ingredients (sucrose, guar gum, sahlep, egg yolk, and skim milk powder) were weighed and added according to formulation presented in Table 1. The prepared mixture was pasteurized at 85°C for 15 min and then quickly cooled to approximately 37 ± 2°C. Subsequently, 1% probiotic culture of *L. acidophilus* DSM 20079 was transferred into ice cream mix and fermented overnight at 37°C. Following the incubation, the BB and JF purees were added separately at a concentration of 1% and 3% (w/w) in ice cream mix (except in the control sample) and mixed homogeneously. Afterward, mixes were aged at 4°C for 24 h and frozen using an automatic horizontal batch freezer (Gürdal Maraşmatik, AG‐30). The ice creams were put into plastic cups (100 mL) and stored at −18°C for 60 days.

**TABLE 1:** Formulations of ice cream.

	Samples				
	Control	1%BB	3%BB	1%JF	3%JF
Probiotic culture (%)	1	1	1	1	1
Milk (mL)	10,000	10,000	10,000	10,000	10,000
Milk powder (g)	1500	1500	1500	1500	1500
Cream (g)	1500	1500	1500	1500	1500
Sucrose (g)	1800	1800	1800	1800	1800
Egg yolk (pcs)	14	14	14	14	14
Sahlep	50	50	50	50	50
Guar gum	30	30	30	30	30
Blueberry puree (%)	–	1	3		
Jujube fruit puree (%)	–	–	–	1	3

Abbreviations: BB, Blueberry; JF, jujube fruit.

### 2.5 Physicochemical analyses

The dry matter (method 941.08), crude ash (method 930.30), and fat (method 952.06) contents of the experimental samples were determined according to the AOAC procedures (AOAC, 2000). These analyses were performed on days 60th of storage at −18°C. Titratable acidity (method 947.05) (AOAC, 2000) and pH values of samples were determined on days 1, 30, and 60 of the storage. A digital pH meter (WTW pH 315i/SET) was used to monitor the pH values of ice cream. The color of the samples was established with a colorimeter (CR‐400 Chroma Meter, Minolta, Japan). The L, a, and b parameters were recorded according to the CIELab color space (McLellan et al., 1995). Color parameters were measured after 1, 30, and 60 days of storage.

### 2.6 Extraction for total phenolic content (TPC) and total antioxidant capacity analyses

For total phenolic and antioxidant activity analyses, ice cream samples extracts were prepared using the modified methods of Özturk et al. (2018). To prepare the extract, 5 g of ice cream was added to 25 mL of 80% methanol solution and homogenized them until uniform consistency with an Ultra‐Turrax®T25 D (IKA, Deutschland, Germany) blender. The mixtures were kept at 25°C for 24 h and centrifuged at 4°C at 7244 g for 10 min and filtered by a membrane filter with a diameter of 0.45 μm before analysis. The sample extracts were kept at 4°C prior to analyses.

### 2.7 TPC and antioxidant activity

TPC of ice creams was measured by Folin–Ciocalteu procedure described in Özturk et al. (2018). TPC was expressed as mg of gallic acid equivalent per g of sample (mg GAE/g). The antioxidant activity was detected by two different methods: radical scavenging capacity, estimated using 2,2‐diphenyl‐1‐picrylhydrazyl (DPPH), as proposed by Brand‐Williams et al. (1995), and ferric reducing antioxidant capacity (FRAP), as described the modified method of Yang et al. (2016).   For the determination of DPPH radical scavenging activity, 100 μL of extraction sample was mixed with 2 mL of 100 μM methanolic solution of DPPH and 900 μL of Tris‐HCl buffer (pH 7.4). The mixture was incubated in darkness at room temperature (25 ± 1°C) for 30 min and the absorbance of sample was measured at 517 nm. DPPH radical scavenging capacity of each sample was explicated as % inhibition. For the FRAP analysis, 75 μL sample extract was mixed with 2.25 mL FRAP reagent and 225 μL distilled water. The absorbance was read at 593 nm after allowing the mixture to incubate in the dark for 30 min at room temperature (25 ± 1°C).

### 2.8 Texture profile analysis (TPA)

TPA was conducted by a texture analyzer (model TA‐XT2, Stable Micro Systems, UK) equipped with a stainless steel cylindrical plate (diameter of 20 mm) and a 50 kg load cell using modified method described in previous work Çam et al., 2023. The condition for TPA: trigger type of auto trigger force of 5 g, test distance of 15 mm, pre‐test speed of 3 mm/s, test speed of 3.3 mm/s, and post‐test speed of 3 mm/s. In texture profile analysis, adhesiveness, gumminess, springiness, hardness, and cohesiveness parameters were determined.

### 2.9 Overrun

The overrun level of experimental samples was determined as described by Sert and Mercan (2021). The results were calculated using the formula below.

$$\text{Overrun}\;\left(\%\right)=\frac{\text{Volume of}\;\mathrm{ice}\;\text{cream}-\text{Volume of}\;\mathrm{mix}}{\text{Volume of}\;\mathrm{mix}}x\;100.$$

### 2.10 Melting test

Ice creams were stored at −18°C before determination of melting behaviors. Ice cream samples (40 g) were put on a metal wire mesh (2.5 mm pore size) and allowed to stand for melting at 24 ± 2°C. The complete melting time (min) and first dripping time (min) were detected, and the weight of the melted portion was noted every 5 min until all samples passed through the screen. The time (min) was plotted against the dripped weight (g) and the slope of melting curve was taken melting rate (g/min) (Muse & Hartel, 2004).

### 2.11 Viability of *L. acidophilus* DSM 20079

The viable count of *L. acidophilus* DSM 20079 was detected after 1, 30, and 60 days of storage at −18°C. For this purpose, 10 g sample was diluted with 90 mL of 0.1% sterile ringer water and serially diluted. MRS (deMan Rogosa and Sharpe) agar (Merck, Darmstadt, Germany) enriched using bromocresol green solution (0.2%, w/v, autoclaved at 121°C for 15 min, 20 mL/L) and clindamycin (5 mg/100 mL, filter‐sterilized, 2 mL/L) plates were used for the selective enumerating of *L. acidophilus* DSM 20079 under anaerobic conditions at 37°C for 48 h, and viable counts of probiotics were calculated as log CFU/g (Darukaradhya et al., 2006). Enumerations of *L. acidophilus* DSM 20079 were carried out in duplicate at 1, 30, and 60 days of storage time.

### 2.12 Sensory analyses

Seven trained panelists from the Department of Food Technology at Selcuk University assessed the sensory properties of ice creams. Randomly coded ice cream samples were served and tested immediately after withdrawing the cups from the freezer. The color–appearance, texture, taste–aroma, melting resistance, mouthfeel, and overall acceptability of probiotic ice creams were assessed on a scale from 1 (for dislike) to 5 (for most liked) points (Çam et al., 2023). After 1 day of frozen storage, all sensory evaluations were conducted.

### 2.13 Statistical analysis

Statistical analysis was conducted using Minitab software (version 16, State College, USA) program. The data were analyzed by one‐way ANOVA and the mean values were compared using the Tukey test at *p* < .05 significance levels.

## 3 RESULTS AND DISCUSSION

### 3.1 Physicochemical properties

The chemical compositions of probiotic ice cream samples are presented in Table 2. The dry matter contents of experimental samples were ranged from 37.33% to 35.46% on the 60th day of storage. Control sample and ice creams supplemented with 1% fruit concentrations presented statistically similar results (*p* > .05). Increasing amounts of BB and JF resulted in decreased dry matter content of ice cream samples. This could be explained by the high moisture contents in BB (85.67%) (Wenchao Liu et al., 2021) and JF (JF powder reconstituted in 88% moisture content). Accordingly, added fruit purees may have shown the dilution effect. In parallel with our observations, Sayar et al. (2022) noted that the total solid content of ice cream decreased significantly with increasing blueberry concentration.

**TABLE 2:** Dry matter, ash, and fat contents of the probiotic ice creams on the 60th day of storage.

Samples	Dry matter (%)	Ash (%)	Fat (%)
Control	36.59 ± 0.00AB	1.30 ± 0.14A	6.20 ± 0.00A
1%BB	37.33 ± 0.26A	1.50 ± 0.14A	5.60 ± 0.28A
3%BB	35.46 ± 0.18C	1.50 ± 0.14A	5.55 ± 0.21A
1%JF	36.62 ± 0.16AB	1.50 ± 0.14A	5.50 ± 0.14A
3%JF	35.63 ± 0.44 BC	1.40 ± 0.00A	5.50 ± 0.14A

Abbreviations: BB, Blueberry; JF, jujube fruit.

*Note*: Different letters in the same column indicate significantly different (*p* < .05).

As seen in Table 2, the ash and fat contents of probiotic ice creams ranged from 1.30% to 1.50% and 6.20% to 5.50%, respectively, on the 60th day of storage. A slight increase was detected in the ash content of ice creams with BB and JF compared to the control sample, but this increment was not significantly different between samples (*p* > .05). When the ash contents of JF and BB were considered, it was seen in the literature that the ash content of JF (2.26%–3.01%) (Li et al., 2007) was higher than that of BB (1.80%) (Reque, Steffens, Jablonski, et al., 2014). However, due to the low level of BB and JF added to the ice cream, it is thought that there was no significant difference between the ash contents of the samples. In contrast to our results, Kotan (2018) reported that the ash content in ice cream slightly decreased with the increment BB concentration, but this diminishment was not statistically significant. Additionally, the slight decline in fat value due to the addition of both fruit purees was not statistically significant (*p* > .05).

The results of titratable acidity and pH of ice creams are demonstrated in Table 3. While the incorporation of both BB and JF into the ice creams slightly reduced the titratable acidity compared to the control sample, these reductions observed depending on the formulations of the samples were not statistically significant. Similar behavior in titratable acidity of probiotic ice creams containing blue myrtle pulps was stated by Özturk et al. (2018). In contrast, the acidity values observed in this study did not agree with Sayar et al. (2022), who found that the acidity value of ice creams with BB was higher than the control sample and these values increased in parallel with the BB concentration. The titratable acidity in control samples exhibited a significant decrease from 0.45% to 0.36% during 60 days of storage, and similarly, this value decreased dramatically in 3%JF sample (*p* < .05). The pH values were determined in the range of 5.86–6.10 during 60 days of storage (Table 3). The pH values did not present a regular increase or decrease depending on the addition of both fruit purees to the ice creams, and in general, the pH of all samples increased compatibly with the decrease in titration acidity as the progressed storage time.

**TABLE 3:** Titratable acidity and pH values of the probiotic ice creams during storage.

	Samples	Storage time (day)		
		1	30	60
Titratable acidity (%)	Control	0.45 ± 0.02A,a	0.41 ± 0.01A,ab	0.36 ± 0.01A,b
	1%BB	0.44 ± 0.02A,a	0.37 ± 0.01A,a	0.40 ± 0.05A,a
	3%BB	0.46 ± 0.02A,a	0.38 ± 0.02A,a	0.36 ± 0.03A,a
	1%JF	0.41 ± 0.00A,a	0.38 ± 0.01A,a	0.34 ± 0.04A,a
	3%JF	0.42 ± 0.00A,a	0.38 ± 0.01A,a	0.32 ± 0.02A,b
pH	Control	5.88 ± 0.02B,b	6.01 ± 0.00C,a	6.10 ± 0.01AB,a
	1%BB	5.92 ± 0.02AB,c	6.01 ± 0.00D,b	6.08 ± 0.10A,a
	3%BB	5.86 ± 0.00B,b	5.95 ± 0.00E,a	5.98 ± 0.04C,a
	1%JF	5.94 ± 0.01A,b	6.03 ± 0.00B,a	6.02 ± 0.02ABC,a
	3%JF	5.91 ± 0.00AB,b	6.03 ± 0.00A,a	6.00 ± 0.01 BC,a

Abbreviations: BB, Blueberry; JF, jujube fruit.

*Note*: ^A‐B^Uppercase letters demonstrate differences between the ice creams in the same storage period, and ^a‐b^lowercase letters represent the differences between the storage times of ice creams (*p* < .05).

The results describing color values of the ice creams over storage are presented in Table 4. As expected, ice cream samples supplemented with BB or JF had significantly lower L* values than the control and the decrease in L* value was the greatest in ice cream with BB. The decrease in L* value demonstrates the darker color of the BB puree. According to the literature, the L* value for raw blueberries is declared to be 33.2 (Nowak et al., 2019) and for fresh and pulp of JF 37.98 and 62.24 (Moradinezhad & Dorostkar, 2021), respectively. In this context, the color of the fruit was reflected in the samples. Similar to our result, the studies reported that the L* values decreased with the supplementation of fruits (Akalın et al., 2018; Vital et al., 2018). The brightness (L*) values in all probiotic ice cream decreased with progress of storage days which was related to the declined overrun values. Lower air in the ice cream results in lower brightness because the air in the ice cream dilutes the color (Goff & Hartel, 2013). Similarly, Çam et al. (2023) stated that the brightness of probiotic ice creams supplemented with pea pod powder decreased from day 1 to day 60 of frozen storage.

**TABLE 4:** The color parameters of the probiotic ice creams during storage.

Color parameters	Samples	Storage time (day)		
		1	30	60
L*	Control	89.53 ± 0.21A,a	91.27 ± 0.11A,a	84.13 ± 0.71A,b
	1%BB	83.09 ± 0.50C,b	86.24 ± 0.24C,a	80.27 ± 0.41C,c
	3%BB	79.10 ± 0.20D,b	82.73 ± 0.34D,a	75.77 ± 0.13D,c
	1%JF	87.53 ± 0.13B,b	91.14 ± 0.30A,a	83.82 ± 0.21AB,c
	3%JF	86.51 ± 0.28B,b	88.85 ± 0.35B,a	82.40 ± 0.33B,c
a*	Control	−3.67 ± 0.04E,a	−3.38 ± 0.08E,a	−4.73 ± 0.13E,b
	1%BB	−1.37 ± 0.22B,a	−1.13 ± 0.05B,a	−2.32 ± 0.01B,b
	3%BB	−0.30 ± 0.04A,a	−0.55 ± 0.22A,a	−0.64 ± 0.08A,a
	1%JF	−3.15 ± 0.02D,b	−2.71 ± 0.04D,a	−3.83 ± 0.16D,c
	3%JF	−1.99 ± 0.17C,a	−1.79 ± 0.08C,a	−2.99 ± 0.08C,b
b*	Control	8.97 ± 0.13B,b	9.22 ± 0.17B,b	11.96 ± 0.05A,a
	1%BB	4.58 ± 0.76C,b	4.24 ± 0.10C,b	8.23 ± 0.05A,a
	3%BB	3.84 ± 0.48C,a	2.79 ± 0.39D,a	9.67 ± 4.47A,a
	1%JF	10.07 ± 0.10AB,b	10.01 ± 0.06B,b	13.224 ± 0.55A,a
	3%JF	10.87 ± 0.33A,b	11.21 ± 0.11A,b	13.93 ± 0.45A,a

Abbreviations: BB, Blueberry; JF, jujube fruit.

*Note*: ^A‐D^Uppercase letters demonstrate differences between the ice creams in the same storage period, and ^a‐c^lowercase letters represent the differences between the storage times of ice creams (*p* < .05).

The negative values for the a* parameter refer to the greenness. The lowest a* value detected in the control sample indicated that this sample had the lowest greenness. The addition of BB caused the highest increase on a* value. The highest positive values of coordinate *b* refer to the highest yellow color. While the b* value was detected to be the highest in the sample 3%JF with a value of 10.87 after 1 day of storage and ice cream supplemented with BB presented the lowest yellowness. This reduction demonstrates that the samples with BB become more bluish in color that cannot be differentiated with eyes. The differences in color parameters of ice creams might be ascribed to the composition of both fruits. Blueberry contains more anthocyanins and flavonoids than jujube fruit (Li et al., 2016; Zhang et al., 2010). Anthocyanins are responsible for the purple color and flavonoids for the blue and red colors (Moyer et al., 2002). Similar findings were seen in previous studies (Goraya & Bajwa, 2015; Ürkek et al., 2019). Moreover, at the end of storage, the yellowness value of all samples increased statistically significant. Freezing treatment reduces blueness and increases yellowness in berry fruits (Nowak et al., 2019). This could be attributed to the conversion of anthocyanins to colorless carbinol bases such that bluish‐brown co‐pigments dominate the fruit color (Stojanovic & Silva, 2007).

### 3.2 TPC and antioxidant activity

**FIGURE 1:**
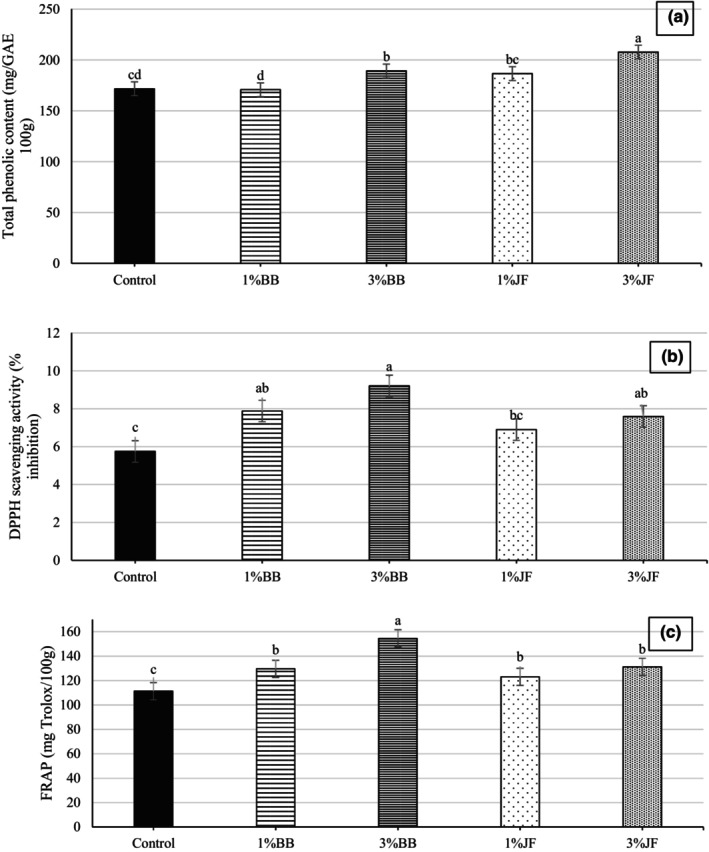
Total phenolic content (a), DPPH (b), and FRAP (c) assays for antioxidant activity of the probiotic ice creams on the 60th day of storage. BB, Blueberry; JF, jujube fruit. ^A‐D^Different letters indicate significantly different (*p* < .05).

TPC and antioxidant activity of the samples on the 60th day of storage are shown in Figure 1. The results showed that the incorporation of BB and JF in ice cream significantly improved the TPC of samples (*p* < .05). The fortification of ice cream with JF caused a higher increase in TPC than that of BB. Also, the TPC of the samples increased dramatically with increasing JF concentration (*p* < .05). In literature, the total phenolic contents of JF are reported as 25–42 mg GAE/g (Kamiloglu et al., 2009) and 42.6–55.4 mg GAE/g weight (Xue et al., 2009). For blueberry, this value is informed to be 2.5–3.1 mg GAE/g (Giovanelli & Buratti, 2009) and these data are supported by those reported by other studies (Lee et al., 2004; Sellappan et al., 2002). As seen in our results, the TPC of fruits affected the TPC of the ice creams and the highest TPC was determined at 3%JF (Figure 1a).

Figure 1b,c demonstrates that the values of antioxidant activity, determined by DPPH and FRAP, are compatible with each other. The antioxidant activity in the ice creams was significantly increased by adding both fruits (*p* < .05). JF and BB are the most important source of bioactive phytochemicals having antioxidative properties, such as ascorbic acid, phenolics, flavonoids (especially anthocyanins), and triterpenes (Kou et al., 2015; Reque, Steffens, Silva, et al., 2014). DPPH and FRAP values varied in the range of 5.75%–9.20% and 111.25–154.40 mg Trolox/100 g in probiotic ice cream. Ice creams with 3%BB showed the highest DPPH and FRAP values with 9.20% and 154.40 mg Trolox/100 g, respectively. In fact, the antioxidant activity results contradict the outcome that the samples with JF contain higher phenolic compounds than those with BB. As reported in previous works, BB has higher anthocyanin and lower TPC than JF (Giovanelli & Buratti, 2009; Li et al., 2016; Xue et al., 2009; Zhang et al., 2010). In our results, the antioxidant content and TPC of the samples were not correlated. This could be associated with the interaction of casein and whey protein with phenolics which had a negative effect on the higher antioxidant activity of phenolic compounds (Mehanna et al., 2014). Contrary to our results, Hwang et al. (2009) stated that the TPC and DPPH scavenging activity results in ice cream supplemented with grape wine lees showed a compatible course with each other.

### 3.3 Texture profile

**FIGURE 2:**
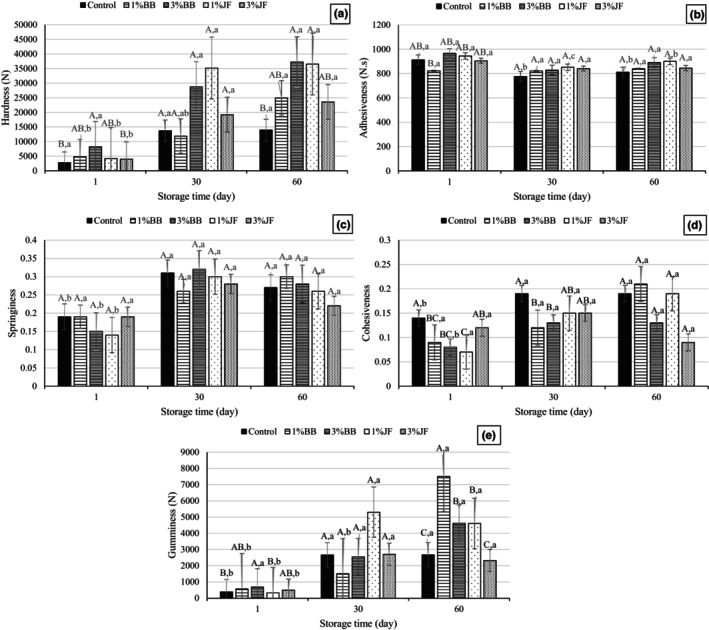
Textural parameters of the probiotic ice creams during storage; hardness (a), adhesiveness (b), springiness (c), cohesiveness (d), gumminess (e). BB, Blueberry; JF, jujube fruit, ^A‐C^Uppercase letters demonstrate differences between the ice creams in the same storage period, and ^a‐b^lowercase letters represent the differences between the storage times of ice creams (*p* < .05).

The TPA parameters of the ice cream samples during the 60 days of storage are presented in Figure 2. To determine the textural properties of ice creams, hardness, cohesiveness, adhesiveness, gumminess, and springiness values were evaluated. Figure 2a shows that the hardness values of the probiotic ice creams ranged from 2760 to 4802 N on the first day of storage. The addition of BB and JF in ice cream caused higher hardness values compared to that of the control. Likewise, Di Criscio et al. (2010) stated that ice cream with added fruit and *Lacticaseibacillus casei* exhibited higher firmness than ice cream with vanilla. The highest hardness value was observed in 3%BB sample during all storage time. While increasing BB concentration significantly improved the hardness of ice cream, an opposite pattern to this result was observed for JF. In all samples, the hardness increased significantly as the storage time progressed; this increment could be the result of the decreasing overrun percentages over the storage time. In parallel with our results, El‐Zeini et al. (2016) reported that ice creams with a higher overrun ratio exhibit lower hardness values when fresh or after 14 days of storage. Also, the increase in hardness depending on the storage period could be due to recrystallization of the ice (BahramParvar et al., 2013).

As can be seen from Figure 2b, although the lowest adhesiveness values were observed in 1%BB on the first day of storage, there was no significant difference between other samples for all storage periods. Contrary to our findings, BahramParvar et al. (2013) reported that as the storage time increased, the ice cream samples became more adhesive and attributed this to ice crystal growth and changes in the texture of the samples. Similar to adhesiveness, springiness was not significantly affected by the incorporation of both fruit puree and storage time (*p* > .05) (Figure 2c).

It was determined that the effects of the different addition ratios of BB or JF and frozen storage periods on cohesiveness were found to be statistically significant after 1 day of storage (*p* < .05) (Figure 2d). The highest value for this parameter was detected in the control sample and the lowest was in 1%JF. Although an increase in cohesiveness values of control and 3%BB was recorded with storage time, the difference between the other samples during storage was not statistically significant (*p* > .05).

The addition of BB showed an effect of increasing the gumminess values (Figure 2e). Similarly, Karaman et al. (2014) reported that the gumminess of the ice cream sample advanced with increase in the persimmon puree concentration in the formulation of ice cream. As with the hardness values, the highest gumminess was determined in the 3% BB sample. In our study, probiotic ice creams showed the characteristics of Maraş‐type (hard type) ice cream due to their low overrun values. The characteristic feature of this type of ice cream is the hard and sticky gummy body (Kurt et al., 2016). Therefore, our results agree with this explanation.

### 3.4 The overrun

The result of overrun for the probiotic ice creams during storage is listed in Table 5. Although the incorporation of both BB and JF fruits into probiotic ice creams did not significantly affect on overrun values of samples (*p* > .05), these parameters decreased significantly as the storage time progressed, inversely related to hardness. The low overrun results in an increase the hardness of the ice cream (Sofjan & Hartel, 2004). In the 3% BB sample, which had a lower overrun value than the other ice creams, the gumminess was found to be higher as well as the hardness, as expected. Similar findings were noted in cape gooseberry and jamun pomace added to ice cream by Erkaya et al. (2012) and Shelke et al. (2020), respectively. Overrun values of ice creams (13.39%–14.98%) were determined quite lower compared to those stated in the literature (approximately ranging from 40% to 120%). It can be said that the type of freezer used could cause this result since the discontinuous freezer used in the current study is less efficient in terms of air incorporation than continuous freezers (Akalın & Erişir, 2008). In this context, the ice creams produced were in the Maraş type (hard type) ice cream category. The overrun in this type of ice cream had to be less than 50% according to the Turkish Food Codex (2004). Additionally, total solid content is one of the effective parameters on overrun (Kurultay et al., 2010). On the 60th day of storage, the lowest overrun was detected in the 3% BB with the lowest dry matter content. The decrease in the overruns of fruit‐added ice creams, which gave lower dry matter compared to the control, could be explained by the proportional decrease in milk solids due to the addition of fruit. Milk solids, especially milk proteins, provide ice cream with perfect body, higher overrun, and good texture (Beegum et al., 2022). Similar to our results, it has been reported that the addition of concentrated cactus pear pulp (El‐Samahy et al., 2009) and tomato juice (Kabir et al., 2020) into ice creams reduced overrun values, since the composition of added fruit may have delayed the incorporation of air into the ice cream, resulting in a reduction in the volume of ice cream and overrun (da Silva et al., 2020).

**TABLE 5:** The overrun values (%) of the probiotic ice creams during storage.

Samples	Storage time (day)		
	1	30	60
Control	14.28 ± 0.00A,a	12.33 ± 2.44A,a	13.20 ± 0.41A,a
1%BB	14.98 ± 0.32A,a	10.84 ± 2.67A,a	12.45 ± 0.63AB,a
3%BB	14.74 ± 0.66A,a	8.95 ± 0.00A,b	9.92 ± 0.56B,b
1%JF	14.90 ± 0.87A,a	11.12 ± 0.72A,b	11.56 ± 0.63AB,b
3%JF	13.39 ± 1.26A,a	11.85 ± 2.83A,a	10.82 ± 1.27AB,a

Abbreviations: BB, Blueberry; JF, jujube fruit.

*Note*: ^A‐B^Uppercase letters demonstrate differences between the ice creams in the same storage period, and ^a‐b^lowercase letters represent the differences between the storage times of ice creams (*p* < .05).

### 3.5 The melting properties

Effect of addition of BB and JF purees in probiotic ice cream formulation and storage time on melting behaviors (first dripping time, melting rate, complete melting time) is presented in Table 6. Both fruit puree additions dramatically influenced the first dripping time, and the highest time was observed in the ice cream with JF on the first day of storage. This may be due to some components that have the ability to absorb the water present in both fruits. On the 30th and 60th days of storage, the samples with the longest first dripping time differed. It was observed that the first dripping time, which was determined as 3.97 min in the control samples, increased to 8.74 and 11.75 min in the samples with 3% BB and 3% JF, respectively, on the first day of storage. Similarly, Sayar et al. (2022) reported that the first dripping times of ice cream produced from camel's milk containing blueberry were longer than the control without blueberry. Likewise, Erkaya et al. (2012) notified that as the cape gooseberry fruit content in ice cream samples increased, the first dripping times in the ice creams were prolonged. As seen in Table 6, the advancing storage period caused an extension of the first dripping times. All samples completed melting ranging from 53.53 to 68.50 min and differences among all ice creams and storage times were not statistically significant. Moreover, melting rate values ranged from 8.46 to 13.75 g/min (Table 6). The addition of BB and JF to ice creams did not remarkably affect the melting rate at all storage times (*p* > .05), probably because of the narrow range of overruns obtainable in this batch freezer. The use of batch freezer in the current study did not allow for sufficient increase and differences in overruns (Muse & Hartel, 2004). Similar to our results, Tarakçı and Durak (2020) who investigated ice creams produced using sour cherry, banana, kiwi, blackberry, and raspberry purees stated that neither fruit type nor storage period had a noticeable impact on melting rate.

**TABLE 6:** Melting behavior of the probiotic ice creams during storage.

	Samples	Storage time (day)		
		1	30	60
First dripping time (min)	Control	3.97 ± 0.43C,b	21.63 ± 0.63A,a	20.60 ± 0.84A,a
	1%BB	6.14 ± 0.04C,c	10.27 ± 1.29C,b	15.81 ± 0.59B,a
	3%BB	8.74 ± 0.42B,b	21.78 ± 0.97A,a	23.07 ± 1.44A,a
	1%JF	11.05 ± 0.70A,b	20.51 ± 1.52A,a	13.63 ± 0.73C,b
	3%JF	11.75 ± 0.79A,a	15.72 ± 0.67B,b	11.97 ± 0.76 BC,b
Complete melting time (min)	Control	53.84 ± 3.80A,a	65.00 ± 1.41A,a	64.18 ± 7.06A,a
	1%BB	59.65 ± 0.50A,a	59.88 ± 7.24A,a	60.24 ± 6.73A,a
	3%BB	53.53 ± 7.01A,a	62.64 ± 3.67A,a	68.50 ± 2.12A,a
	1%JF	54.67 ± 6.60A,a	66.38 ± 7.10A,a	58.30 ± 1.60A,a
	3%JF	63.17 ± 18.57A,a	66.00 ± 1.41A,a	58.50 ± 2.90A,a
Melting rate (g/min)	Control	8.92 ± 1.11A,a	12.81 ± 0.73A,a	12.55 ± 1.03A,a
	1%BB	9.00 ± 0.00A,c	11.53 ± 0.43A,b	13.75 ± 0.30A,a
	3%BB	9.76 ± 2.78A,a	13.49 ± 0.67A,a	12.14 ± 0.42A,a
	1%JF	8.66 ± 2.21A,a	12.63 ± 1.33A,a	12.14 ± 0.24A,a
	3%JF	8.46 ± 3.14A,a	11.30 ± 0.54A,a	12.37 ± 0.19A,a

Abbreviations: BB, Blueberry; JF, jujube fruit.

*Note*: ^A‐B^Uppercase letters demonstrate differences between the ice creams in the same storage period, and ^a‐b^lowercase letters represent the differences between the storage times of ice creams (*p* < .05).

### 3.6 Survival of *L. acidophilus* DSM 20079

**FIGURE 3:**
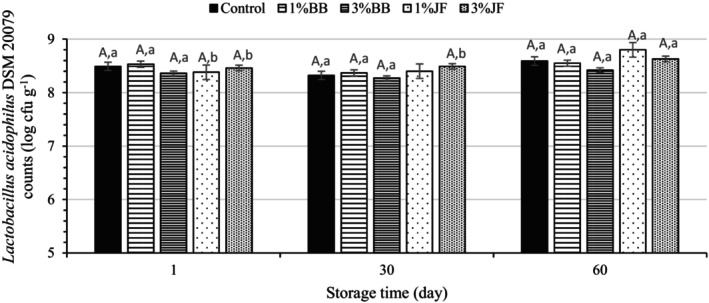
*Lactobacillus acidophilus* DSM 20079 counts (log CFU/g) in the probiotic ice creams during storage. BB, Blueberry; JF, jujube fruit, ^A^Uppercase letters demonstrate differences between the ice creams in the same storage period, and ^a‐b^lowercase letters represent the differences between the storage times of ice creams (*p* < .05).

Figure 3 shows the viability of *L. acidophilus* DSM 20079 in ice creams on 1st, 30th, and 60th days of frozen storage at −18°C. The bacterial counts in the starting culture were 8 log CFU/mL. Viable cell counts of *L. acidophilus* DSM 20079 in the probiotic ice creams ranged between 8.36 and 8.53 log CFU/g 1 day after the freezing process. As seen from the results, the counts of *L. acidophilus* DSM 20079 in the samples increased by 0.2–0.4 log compared to the count of probiotic cells in the inoculum. This indicates that freezing did not have a significantly destructive effect on probiotic cells due to freezing damage of cells. Also, the survival of microorganisms in ice cream is determined by the presence of oxygen, pH, and the components of the mix (Ferraz et al., 2012). The addition of BB and JF did not result in a significant difference between the samples for viable probiotic bacterial counts in all storage times (*p* > .05). The ingredients used in the ice cream formulation may support or adversely affect probiotic viability. Kavas and Kavas (2016) stated that the addition of strawberry and guava pulp improved the *Bifidobacterium* spp. and *Lactobacillus acidophilus* counts in frozen yogurt made with camel milk. Likewise, Özturk et al. (2018) reported that the supplementation of white and dark blue fruit of *Myrtus communis* to probiotic ice creams produced from goat milk increased the counts of *Lacticaseibacillus casei* 431. However, it has also been reported that fortification of ice cream with umbu (*Spondias tuberosa*) pulp reduced commercial cultures (LA‐5 and BB‐12) viability over time (de Oliveira et al., 2021). Considering the trend exhibited by the probiotic counts in the samples on the 1st and 60th days of storage, the number of viable probiotics remained constant in the control and BB added samples, but the probiotic counts increased from 8.38 to 8.80 log CFU/g and from 8.46 to 8.63 log CFU/g in ice cream supplemented with 1 and 3%JF, respectively. This means that JF improved the viable cell count for *L. acidophilus* DSM 20079 in ice cream better than BB as progressed storage time. Moreover, all probiotic ice creams provided the minimum required to achieve probiotic effects after 60 days of frozen storage. Contrary to our results, Sabet‐Sarvestani et al. (2021) stated that the number of probiotics decreased during the storage in symbiotic ice cream using *Lacticaseibacillus casei*/*Lactiplantibacillus plantarum* and fructooligosaccharides, due to the reduction of pH and the freezing shock.

### 3.7 Sensory assessment

**FIGURE 4:**
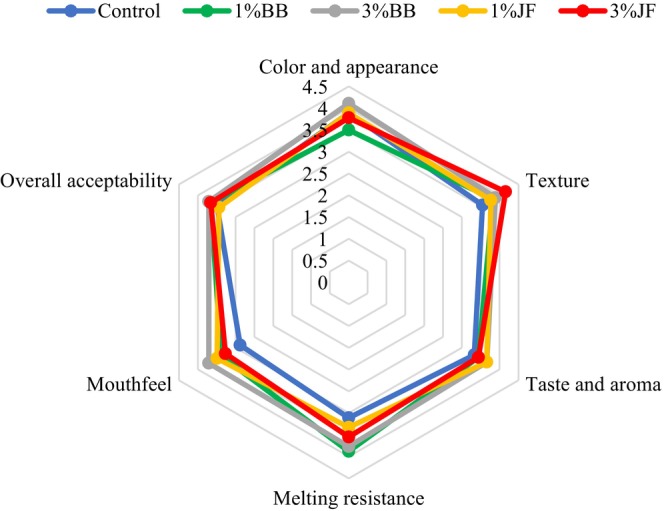
Sensory profile of the probiotic ice creams after 1 day of storage. BB, Blueberry; JF, jujube fruit.

Sensorial properties of probiotic ice cream are profiled in Figure 4. The color– appearance and texture scores were determined in the range of 3.50–4.11 and 3.55–4.16 points, respectively. The most appreciated ice cream was the one with 3%BB for color–appearance and the sample with 3%JF for texture, but they were not statistically significant. This color distinctness was due to the higher a* and lower b* values of ice cream with BB (*p* < .05). Similarly, Özturk et al. (2018) reported that probiotic ice cream with blue myrtle fruit received the most appreciation regarding color and appearance. Also, the highest melting resistance score was found to be 3.88 in %1BB and the lowest score in control with 3.11 point (*p* < .05). However, there were no dramatic differences between sensory parameters scores of all ice cream samples, except for the melting resistance score (*p* > .05). Besides, the BB and JF added ice creams were not perceived as different compared to control ice creams, probably because the flavors were not dominant. The similarity between taste and aroma scores was supported by the results that titratable acidity (Table 3) and *L. acidophilus* DSM 20079 counts (Figure 3) were quite close on the first day of the storing time and the differences between all samples were not statistically significant. Similar results were presented by Kavaz Yuksel (2015), who further stated that the effect of blackthorn (*Prunus spinosa* L.) addition on sensory properties other than color was not statistically significant in ice cream produced with blackthorn at different concentrations. As a result, BB and JF fruit purees can be preferred in probiotic ice cream formulations in terms of consumer acceptance.

## 4 CONCLUSION

In this study, the supplementation of the BB and JF purees increased the DPPH radical scavenging and ferric‐reducing activities, as well as TPC, and improved the functional properties of ice creams. Besides, the highest DPPH and FRAP values were observed in the 3%BB ice cream. The addition of both fruits advanced the hardness and the first dripping time of probiotic ice creams. Adding different levels of BB and JF to ice cream formulations had no significant effect on *L. acidophilus* DSM 20079 counts in ice cream. This result may be attributed to the addition of both fruits after fermentation with *L. acidophilus* DSM 20079 to prevent the color and aroma compounds in the fruits from being affected by the development of acidity in ice cream production. Further research is needed to understand the effect of BB and JF fruits on *L. acidophilus* DSM 20079. However, this probiotic strain showed high viability during storage, maintaining the bacterial count of 10^7^–10^8^ CFU/g recommended by the International Dairy Federation for probiotic products. Also, the sensory evaluation results showed that neither the addition of BB nor JF to probiotic ice creams affected consumer preferences. Consequently, BB and JF fruit purees can be added at higher concentrations in the formulations of probiotic ice cream in future studies and a significant difference may be made in terms of acceptability by the consumer.

## AUTHOR CONTRIBUTIONS

**Gülsüm Şentürk:** Data curation (equal); formal analysis (equal); investigation (equal); methodology (equal). **Nihat Akın:** Funding acquisition (equal); project administration (equal); resources (equal); supervision (equal). **Çiğdem Konak Göktepe:** Conceptualization (equal); formal analysis (equal); investigation (equal); software (equal); validation (equal); writing – original draft (equal); writing – review and editing (equal). **Begüm Denktaş:** Formal analysis (equal); investigation (equal).

## CONFLICT OF INTEREST STATEMENT

The authors declare that they have no conflict of interest.

## Data Availability

The data that support the findings of this study are available from the corresponding author upon reasonable request.
